# Impact of experimental hypercalcemia on routine haemostasis testing

**DOI:** 10.1371/journal.pone.0175094

**Published:** 2017-03-31

**Authors:** Giuseppe Lippi, Gian Luca Salvagno, Giorgio Brocco, Matteo Gelati, Elisa Danese, Emmanuel J. Favaloro

**Affiliations:** 1 Section of Clinical Biochemistry, University of Verona, Verona, Italy; 2 Department of Haematology, Sydney Centres for Thrombosis and Haemostasis, Institute of Clinical Pathology and Medical Research, NSW Health Pathology, Westmead Hospital, Westmead, New South Wales, Australia; Institut d'Investigacions Biomediques de Barcelona, SPAIN

## Abstract

**Background:**

The blood to anticoagulant ratio is standardized according to the physiological calcium concentration in blood samples conventionally used for hemostasis testing. Specifically, one fixed volume of 0.109 mmol/L sodium citrate is added to 9 volumes of blood. Since little is known about the impact of hypercalcemia on the calcium-binding capacity of citrate, this study was planned to investigate the effect of experimental hypercalcemia on routine hemostasis testing.

**Methods:**

Fifteen pooled citrated plasmas with matching lithium-heparin pooled plasma from patients with different values of prothrombin time (PT) were divided in three aliquots of 0.6mL each. The first paired aliquots of both citrate and lithium-heparin plasma were supplemented with 60μL of saline, the second paired aliquots with 30μL of saline and 30μL of calcium chloride and the third paired aliquots with 60μL of calcium chloride. Total and ionized calcium was measured in all aliquots of citrate and lithium-heparin plasma, whereas PT, activated partial thromboplastin time (APTT) and fibrinogen were measured in citrate plasma aliquots.

**Results:**

Total calcium concentration gradually increased in both lithium-heparin and citrate plasma aliquots 2 and 3 compared to baseline aliquot 1. The concentration of ionized calcium also gradually increased in lithium-heparin plasma aliquots 2 and 3, whereas it remained immeasurable (i.e., <0.10 mmol/L) in all citrate plasma aliquots. No significant differences were observed for values of PT, APTT and fibrinogen in citrate plasma aliquots 2 and 3 compared to the baseline aliquot 1, with a mean bias was always comprised within the desirable quality specifications derived from biological variability data.

**Conclusion:**

Hypercalcemia, up to severe hypercalcemia does not generate significant bias in results of first-line coagulations tests, so that hypothetical consideration of adjusting citrate-blood ratio is unjustified in hypercalcemic patients.

## Introduction

Laboratory testing is vital for screening, diagnosing and monitoring treatment related to hemostasis disturbances, as associated with both thrombotic and bleeding risk [1,2]. Coagulation tests are usually classified according a conventional hierarchy of complexity, entailing first-line (i.e., screening), second-line (i.e., diagnostic) and third-line tests, the last of which are usually performed to help defining the specific nature and severity of an underlying hemostatic disorder [3]. Due to the crucial role of haemostasis testing in diagnosing and managing hemostasis disturbances, a high degree of accuracy must be assured throughout the total testing process, i.e., from sample collection to testing and clinical interpretation of data [4–7].

Like other areas of laboratory medicine (in particular, clinical chemistry) there are many preanalytical confounders to accuracy, if not appropriately controlled for or identified. Thus, inaccurate data of coagulation tests may be due to many preanalytical problems, the most important of which include incorrect sample type and potential contamination [8]. As regards to haematocrit, increased values, typically above 0.55 (i.e., >55%), may substantially impair the ratio of blood to citrate in plasma, resulting in severe derangements of coagulation test results. Importantly, the amount of citrate that remains free after ionized calcium in the plasma sample has been bound may then also bind to the same ion upon sample recalcification and before performing coagulation tests, so that any variation in the conventional ratio between calcium and citrate may impair the clotting times of many hemostasis tests [9]. To overcome the problem of high hematocrit, for example, the Clinical and Laboratory Standards Institute (CLSI) document H21-A4 contains specific instructions that citrate concentration should be adjusted for haematocrit values above 0.55 [10], and this can be accomplished by using a simple formula such as: [residual volume of citrate in the tube] = [100-hematocrit]*[sample volume]/[595-hematocrit]. This background highlights that biological factors impairing the standardized blood to citrate ratio may ultimately jeopardize the reliability of coagulation test results. Among these factors, therefore, hypercalcemia may also play an important role.

The current CLSI guidelines recommend that blood should be preferably drawn into evacuated blood tubes containing 0.105–0.109 mol/L (i.e., 3.2%) buffered sodium citrate, and that an accurate blood to anticoagulant ratio of 9:1 should be fulfilled in order to maintain a standardized ratio between the binding capacity of this anticoagulant and the calcium present in the blood sample [10]. The plasma and serum concentration of total calcium is normally comprised between 2.0–2.5 mmol/L, whereas that of ionized calcium is normally comprised between 1.0–1.4 mmol/L. Modest hypercalcemia is then diagnosed when total calcium in plasma or serum is 3.0–3.5 mmol/L, whereas severe hypercalcemia is diagnosed when total calcium in plasma or serum is >3.5 mmol/L [11]. It is feasible that the excess of calcium present in the blood of hypercalcemic patients may not be completely neutralized by the standardized concentration of citrate in the blood tube, potentially leading to spurious alternation of clotting times. Interestingly, nearly 35 years ago, Small and Mallette reported the case of undue clotting of a complete blood cell count blood tube anticoagulated with EDTA, due to the presence of severe hypercalcemia [12], thus confirming that an excess of calcium in blood may overwhelm the binding capacity of conventional anticoagulant additives with calcium-binding capacity. However, no studies have specifically assessed the potential impact of hypercalcemia on coagulation testing to the best of our knowledge. Therefore, this original investigation planned to investigate the impact of experimental hypercalcemia on first-line, routine hemostasis testing, encompassing performance of prothrombin time (PT), activated partial thromboplastin time (APTT) and fibrinogen.

## Materials and methods

A total number of 30 routine coagulation samples were identified according to their PT value, and concomitant availability of a paired lithium-heparin specimen referred for routine calcium measurement. The samples were selected as follows: 10 routine samples with values of PT comprised between 0.97–1.09 (set A; routine ‘normal haemostasis’ samples), 10 samples from patients on oral anticoagulant therapy (OAT) with values of PT comprised between 1.50–2.50 (set B; modestly ‘abnormal’ or ‘anticoagulated’ samples) and 10 samples of patients on OAT with values of PT comprised between 2.5–3.7 (set C; highly abnormal or anticoagulated samples). These were selected in order to assess any potential effect of hypercalcemia on both normal and abnormal samples, including patients being monitored for therapy, and where an effect might affect their clinical management. All routine samples were collected in evacuated blood tubes containing either 0.109 mmol/L buffered sodium citrate (citrate plasma; Vacutest Kima, Padova, Italy) or lithium-heparin (lithium-heparin plasma; Vacutest Kima) and were then separated by standard centrifugation at 1500 g per 10 min at room temperature.

To obtain the minimum amount of plasma volume necessary for testing, 1.0 mL of two citrate patient samples of each set (A; B; C) were pooled to obtain a final volume of 2.0 mL each, thus producing fifteen separate pooled citrate plasmas, five for each set. Each citrate plasma pool was then used to prepare 3 identical aliquots (aliquots 1, 2 and 3) of exactly 0.6 mL. Similarly, 1.0 mL of two lithium-heparin patient samples of each set (A; B; C), exactly matching the citrate plasma specimens used for the previous procedure, were also pooled to obtain a final volume of 2.0 mL, thus producing fifteen lithium-heparin plasmas, five for each set. According to this procedure, then, each lithium-heparin plasma pool was made from the same patient plasmas used to obtain the citrate plasma pools. Each lithium-heparin plasma pool was then used to prepare 3 identical aliquots (aliquots 1, 2 and 3) of exactly 0.6 mL, as per the citrate plasma aliquots, thus producing matched pairs. A stock solution of 0.02 mol/L calcium chloride (HemosIL 0.020 M; Instrumentation Laboratory; Bedford, MA, USA) was used to experimentally increase the calcium concentration of citrate and lithium-heparin plasma. Briefly, 60 μL of saline was added to each aliquot of citrate plasma and lithium-heparin plasma labelled as aliquot 1, 30 μL of saline plus 30 μL of calcium chloride solution were added to each aliquot of citrate plasma and lithium-heparin plasma labelled as aliquot 2, and 60 μL of calcium chloride solution were added to each aliquot of citrate plasma and lithium-heparin plasma labelled as aliquot 3.

The PT, APTT and fibrinogen were measured on plasma citrate aliquots 1, 2 and 3 using an ACL TOP 700 coagulation analyzer (Instrumentation Laboratory), with RecombiPlasTin (Instrumentation Laboratory), SynthASil (Instrumentation Laboratory) and Fibrinogen-CXL (Instrumentation Laboratory), respectively. The analytical performance of these tests has been described elsewhere [13]. The normal PT ratio was calculated as for current indications, on a population of 120 ostensibly healthy blood donors. More specifically, the PT value in seconds of each subject was divided by the mean normal PT in seconds (MNPT; the geometric mean PT value of 20 healthy subjects). The reference range was then calculated to comprise the 95% confidence interval of the population. Total calcium was measured on both citrate and lithium-heparin aliquots 1, 2 and 3 using a Cobas 6000 clinical chemistry analyzer (Roche Diagnostics GmbH, Penzberg, Germany), with the 5-Methyl-5’-nitro BAPTA reagent. Ionized calcium was assayed on both citrate and lithium-heparin aliquots 1, 2 and 3 on GEM Premier 4000 (Instrumentation Laboratory), which uses a calcium-selective electrode. As claimed by the manufacturer, the reportable range of ionized calcium is comprised between 0.10–5.00 mmol/L. All tests were performed in duplicate (results were finally averaged), within 3 hours of sample arrival in the laboratory.

The results of testing are shown as mean and standard deviation (SD), and were analyzed with paired Student’s T-test, Spearman’s correlation and Bland and Altman plots. Significance of percentage variation of coagulation testing in hyperalcemic plasmas was compared with the current desirable quality specifications for bias derived from intra- and inter-individual biologic variation (PT, 2.0%; APTT, 2.3%; fibrinogen, 4.8%) [14]. The statistical analysis was performed using the statistical software Analyse-it (Analyse-it Software Ltd, Leeds, UK). The study was carried out in accordance with the Declaration of Helsinki and was approved by the Institutional Review Board of the University Hospital of Verona (Approval number: 970CESC). In accordance with ‘quality assurance studies’, informed consent was deemed as unnecessary, since the entire study was performed using residual plasma (i.e., routine samples referred for hemostasis and clinical chemistry testing, used for the experiments after routine testing had been completed, and which would otherwise be discarded).

## Results

The main results of this study are shown in Table 1.

**Table 1:** Effect of experimental hypercalcemia on Prothrombin Time (PT), Activated Partial Thromboplastin Time (APTT) and fibrinogen. Results are shown as mean and standard deviation (SD).

Parameter	Aliquot 1	Aliquot 2		Aliquot 3	
	Value	Value	p*	Value	p*
Total calcium in lithium-heparin plasma (mmol/L)	2.18±0.09	3.10±0.10	<0.001	4.12±0.16	<0.001
Ionized calcium in lithium-heparin plasma (mmol/L)	0.87±0.03	1.29±0.09	<0.001	1.60±0.13	<0.001
Total calcium in citrate plasma (mmol/L)	1.89±0.08	2.84±0.09	<0.001	3.79±0.08	<0.001
Ionized calcium in citrate plasma (mmol/L)	<0.10	<0.10	-	<0.10	-
PT (sec)	22.3±10.1	22.3±10.0	0.470	22.4±10.1	0.154
APTT (sec)	31.6±5.6	31.5±5.6	0.127	31.3±6.6	0.141
Fibrinogen (g/L)	3.93±0.79	3.92±0.92	0.459	3.99±0.86	0.170

* versus aliquot 1

**Fig 1:**
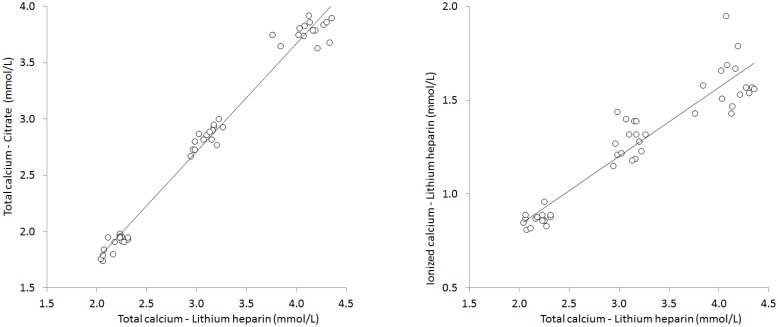
Spearman’s correlation between total calcium in lithium-heparin plasma and total calcium in citrate plasma or ionized calcium in lithium-heparin plasma.

As predictable, the total calcium concentration gradually increased in lithium-heparin and citrate plasma aliquots 2 (lithium-heparin plasma: 3.10±0.10 mmol/L; modest hypercalcemia) and 3 (lithium-heparin plasma: 4.12±0.16 mmol/L; severe hypercalcemia) compared to the baseline aliquot 1 (lithium-heparin plasma: 2.18±0.09 mmol/L; normal calcium concentration). A highly significant correlation was found between total calcium measured in all lithium-heparin and all citrate plasma aliquots (r = 0.991; p<0.001) (Fig 1).

**Fig 2:**
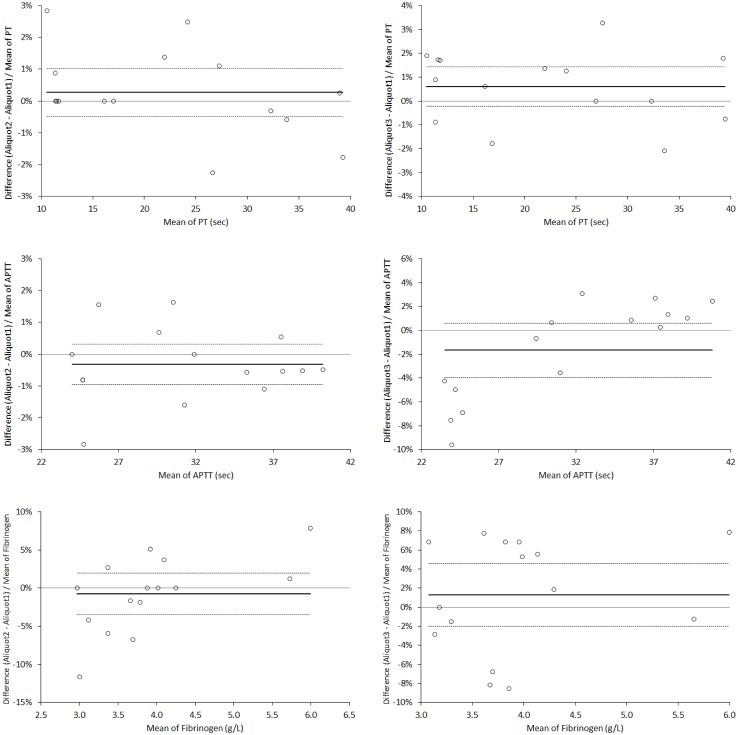
Bland and Altman plots of Prothrombin Time (PT), Activated Partial Thromboplastin Time (APTT) and fibrinogen in paired aliquots of citrate plasma with increasing amount of calcium. The continuous and dotted horizontal lines indicate the mean bias and the 95% confidence interval (95% CI).

The concentration of ionized calcium also gradually and substantially increased in lithium-heparin plasma aliquots 2 (1.29±0.09 mmol/L) and 3 (1.60±1.13 mmol/L) compared to the baseline aliquot 1 (0.87±0.03 mmol/L), whereas it remained always not measurable (i.e., <0.10 mmol/L) in all citrate plasma aliquots 1, 2 and 3. A highly significant correlation was also observed between total and ionized calcium measured in all lithium-heparin aliquots (r = 0.941; p<0.001) (Fig 1). As regards to results of coagulation testing, no significant differences were seen for the values of PT, APTT and fibrinogen in citrate plasma aliquots 2 and 3 compared to the baseline aliquot 1 (Table 1). Accordingly, the mean bias estimated with Bland and Altman plot analyses was always comprised within the desirable quality specifications (Fig 2).

More specifically, when compared to the baseline aliquot 1, the bias was 0.3% (95% CI, -0.5% to 1.0%) in aliquot 2 and 0.6% (95% CI, -0.2% to 1.4%) in aliquot 3 for PT, -0.3% (95% CI, -1.0% to 0.3%) in aliquot 2 and -1.7% (95% CI, -4.0% to 0.6%) in aliquot 3 for APTT, -0.8% (95% CI, -3.5% to 2.0%) in aliquot 2 and 1.3% (95% CI; -2.0% to 4.6%) in aliquot 3 for fibrinogen, respectively.

## Discussion

The first evidence that a concentration of 0.01 mmol/L of sodium citrate may be sufficient to inhibit the coagulation process by neutralizing a physiological concentration of ionized calcium was provided by Quick and Stefanini nearly 70 years ago [15]. Since then, only few studies have been published regarding the potential impact of hypercalcemia on citrate-binding capacity and hemostasis testing. More than 40 years ago, Hilgard evaluated the effect of experimental hypercalcemia on blood coagulation in mice [16]. Hypercalcemia was induced by transplanting solid Walker 256 cancer and intraperitoneally injecting calcium gluconate. The effect on blood coagulation was then assessed by measuring whole blood clotting times in polystyrene and glass test tubes. A significant shortening of clotting times was observed at serum calcium concentrations between 5.1–5.7 mmol/L, thus being virtually incompatible with life, which was also found to be more pronounced in polystyrene (-44%) than in glass (-25%) tubes. Additional data suggesting that calcium may actually influence blood coagulation was conveyed by Bristow et al, who evaluated the impact of calcium supplements on thromboelastography (TEG) in post-menopausal women [17]. Interestingly, an increase of coagulation index was only noticed 4 h after ingestion of 1 g of calcium citrate, yielding a significant shortening of the time of clot initiation, thus reflecting a greater tendency toward hypercoagulability. No effects were instead observed at other different time points (i.e., 2, 6 and 8 hours).

The use of buffered sodium citrate anticoagulation for blood in laboratory diagnostics is unavoidable, since this additive generates a stable source of both blood and plasma then used for many haemostasis tests. As mentioned, the volume of citrate in evacuated blood tubes is standardized according to a constant volume of blood (typically 1:9), but this standard does not consider potential changes to the concentration of calcium in human blood, which may be occasionally increased, for example in patients with hypercalcemia, which may in turn arise for many reasons (e.g., hyperparathyroidism, cancer, granulomatous diseases, immobilization, increased intake or absorption and use of thiazide diuretics). Despite recognition that high hematocrit or insufficient filling of evacuated blood tubes are frequent causes of spurious prolongation of both PT and APTT for decades [18], very little attention has been given to the fact that calcium in blood or plasma is actually the main target of citrate anticoagulation, so that an excess of this ion may overwhelm the calcium-binding capacity of citrate. The results of our study clearly attest, however, that neither mild or severe hypercalcemia generate a substantial bias in test results of PT and APTT, so that the use of corrective formulas for adjusting the ratio between blood and citrate in patients with calcium concentration exceeding the upper limit of the normal range (i.e., >2.5–2.6 mmol/L) is neither necessary or advisable. Notably, the concentration of ionized calcium in all citrate aliquots in our study, including those with a corresponding concentration of 1.60±0.13 mmol/L in lithium-heparin plasma (i.e., aliquots 3), were always immeasurable (i.e., <0.10 mmol/L), thus suggesting that the actual concentration of 0.109 mol/L buffered sodium citrate, as conventionally used for anticoagulation of plasma for coagulation studies, is sufficient to sequestrate all the excess of ionized calcium even in hypercalcemic patients, displaying total and ionized calcium concentrations as high as 4.1 mmol/L and 1.6 mmol/L, respectively. This is consistent with previous data showing that the probability of clot formation is extremely unlikely below a concentration of 0.23 mmol/L of ionized calcium [19], whereas normal clotting in recalcified plasma can only be attained in citrate plasma samples in which the concentration of ionized calcium exceeds 0.56 mmol/L [20].

Despite the limited number of samples tested and the in vitro nature of this study, we hence conclude that hypercalcemia, inclusive of severe hypercalcemia (i.e., total calcium values >3.0 mmol/L and ionized calcium values >1.40 mmol/L) does not generate a significant bias in results of first-line, routine coagulations tests.
